# Sedation in Traumatic Brain Injury

**DOI:** 10.1155/2012/637171

**Published:** 2012-09-20

**Authors:** Oliver Flower, Simon Hellings

**Affiliations:** ^1^University of Sydney, Sydney, NSW, Australia; ^2^Department of Intensive Care, Royal North Shore Hospital, Sydney, NSW 2065, Australia

## Abstract

Several different classes of sedative agents are used in the management of patients with traumatic brain injury (TBI). These agents are used at induction of anaesthesia, to maintain sedation, to reduce elevated intracranial pressure, to terminate seizure activity and facilitate ventilation. The intent of their use is to prevent secondary brain injury by facilitating and optimising ventilation, reducing cerebral metabolic rate and reducing intracranial pressure. There is limited evidence available as to the best choice of sedative agents in TBI, with each agent having specific advantages and disadvantages. This review discusses these agents and offers evidence-based guidance as to the appropriate context in which each agent may be used. Propofol, benzodiazepines, narcotics, barbiturates, etomidate, ketamine, and dexmedetomidine are reviewed and compared.

## 1. Introduction

Several different classes of drugs are used as sedatives in patients with traumatic brain injury (TBI). Several of these agents may also have other uses, for example as anticonvulsants or analgesics. Whilst none are perfect, they all have potential roles in managing a condition that is a major cause of disability, death, and economic cost to society. This paper discusses and compares these agents and offers evidence-based guidance as to the appropriate context in which each agent may be used.

It is important to delineate the contexts in which sedative agents are used in the setting of TBI and what is considered a sedative. For the purposes of this paper, sedative agents are considered to be drugs that decrease consciousness and have therapeutic applications in the management of TBI. After primary brain injury, airway protection and control of ventilation are often required. Induction sedative agents (distinct from muscle relaxants) are used to safely facilitate endotracheal intubation in a manner that minimises haemodynamic instability and secondary brain injury. Maintenance of sedation is then employed as part of the overall management of TBI to permit manipulation of ventilation, optimisation of cerebral metabolic rate (CMRO_2_), cerebral blood flow (CBF), and intracranial pressure (ICP). See Table 1 for abbreviations with explanations. In TBI confounded by alcohol or illicit drug intoxication, sedative agents facilitate safe management whilst these confounding drugs wear off. For refractory, elevated ICP in severe TBI, sedative agents play a key role in the escalating tiers of therapy to reduce ICP. Sedative hypnotics are also employed in seizure control for refractory acute posttraumatic epilepsy. As with all ventilated patients, sedatives act as anxiolytics whilst patients are mechanically ventilated [1].

The primary injury of TBI causes diffuse axonal injury, cerebral oedema, intracranial haematoma, elevated ICP, reduced cerebral perfusion pressure (CPP), and cerebral ischaemia. Therapeutic efforts focus on reducing the secondary insults of hypoxia, hypercapnea, systemic hypotension and intracranial hypertension. Sedatives address these issues in several ways. They allow optimisation of ventilation to prevent hypoxia and achieve normocapnea (and hypocapnea for brief episodes of elevated ICP); they reduce CMRO_2_ and therefore CBF and cerebral blood volume (CBV) and reduce ICP. However they may reduce systemic blood pressure, thereby reducing CPP, and have other adverse effects. Even a single episode of hypotension is a powerful predictor of outcome following TBI [2, 3].

There is limited evidence available to guide the choice of specific sedative agents in TBI. A recent systematic review examining a range of outcomes in TBI concluded that there was no convincing evidence that any one of the sedative agent was superior to another [4]. A number of these studies included patients with less severe traumatic brain injuries and spanned several decades, further limiting conclusions that can be made. Multiple sedative agents are often used synchronously, clouding assessment of individual agents. The guidelines from the Brain Trauma Foundation also highlight a lack of high quality evidence to recommend one sedative agent over another, with the exception of barbiturate use for refractory elevated ICP. Despite this, each agent has a potential role in TBI and clinicians must consider the advantages and disadvantages when deciding what to use in each context.

## 2. Propofol

See Table 2. Propofol is a phenol derivative with high lipid solubility and a rapid onset of action. It has a very low solubility in water so is formulated as an emulsion in soya bean oil, glycerol, and egg phosphatide. A relatively rapid plasma clearance ensures a reliable recovery of consciousness even after prolonged administration, thereby facilitating neurological examination. However, the context-sensitive half time does increase with prolonged infusions, though to a much lesser extent than seen with many other sedatives.

Since its introduction in 1986, propofol has increasingly been used both as an induction agent and as a maintenance sedative in the neurointensive care unit. Several studies have demonstrated the favourable cerebral effects of propofol. ICP, CBF, and CMRO_2_, have all been shown to be reduced with propofol [8, 9]. However, a fall in mean arterial blood pressure (MAP) may reduce the CPP if this is not mitigated with adequate fluid resuscitation and vasopressors. When comparing propofol sedation with midazolam in medical and surgical ICU patients, propofol has been associated with improved quality of sedation and a faster recovery of consciousness on discontinuation of sedation [10].

There is increasing awareness in the literature of the central role of mitochondrial dysfunction and cerebral cell death in areas of the brain with high oxidative stress [11, 12]. Propofol may act as a neuroprotective agent through limitation of oxidative stress. An RCT employing cerebral microdialysis catheters compared sedation with midazolam and propofol, using several cerebral biomarkers as endpoints in the acute phase of TBI [13]. No difference was found between the two groups over a 72-hour period in the lactate to pyruvate ratio, a marker of cerebral oxidative stress. This was a relatively small study and the concentrations of propofol used may not have been sufficient to produce an antioxidant effect nevertheless this is an interesting and novel area of future research.

**Figure 1:**
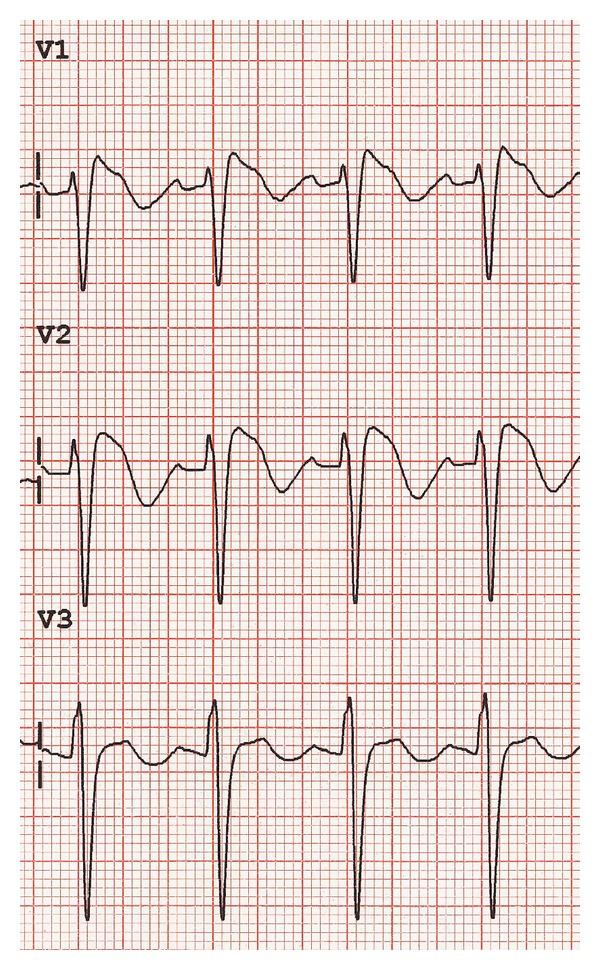
Brugada-like ECG changes that may be seen in propofol infusion syndrome. Coved ST elevation, at least 2 mm J point elevation and descending ST segment followed by a negative T wave (see [67]).

Aside from a reduction in MAP and the need for increased vasopressor requirements to preserve CPP, the lipid formulation of propofol may be associated with other adverse effects. Propofol infusion syndrome (PRIS) was initially described in case studies of children who were sedated with propofol infusions. Subsequently it has been reported in adults, both with long-term infusions in ICU patients and in the short term when used as a general anaesthetic. Clinically patients may present with a variety of findings including lactic acidosis, cardiac dysfunction, and Brugada-like electrocardiogram changes (see Figure 1), which may herald imminent malignant arrhythmias [14]. This can progress to rhabdomyolysis, renal failure, and cardiovascular collapse. The pathophysiology of PRIS is incompletely understood and involves multiple different pathways. An underlying imbalance between energy utilization and demand at the mitochondrial level and effects on lipid metabolism are postulated mechanisms.

Importantly, it is thought that PRIS is more common in patients with TBI. In one retrospective cohort study of adult neurosurgical patients in ICU, 7 of 67 patients displayed signs of PRIS and died. There was an increased incidence of PRIS with higher doses [15]. PRIS may be more common in TBI because large doses of propofol can be used to control elevated ICP [16]. It has been argued that PRIS may limit the usefulness of propofol as a sedative agent in traumatic brain injury, particularly when used in higher doses.

Other potential complications associated with the use of propofol include an elevation in pancreatic enzymes and pancreatitis [17]. Concerns have also been raised that propofol offers a good medium for microbial growth [18], although this may be less significant with newer formulations. Propofol has a significant calorific content, and this should be taken into account when performing nutritional assessments.

Initial reports suggested that propofol may increase seizure activity in susceptible patients [19]. The extent to which this activity represented disordered muscle tone or true seizure activity is unclear [20]. Conversely, propofol has also been demonstrated to increase seizure threshold and has been successfully used in the treatment of status epilepticus. Much of the evidence for the use of propofol in refractory status epilepticus is derived from case series that demonstrated cessation of seizure activity with infusions of propofol [21]. Propofol has been demonstrated to achieve and maintain burst suppression, although at the expense of significant decreases in mean arterial pressure and cardiac index [22].

Therefore, propofol is indicated as a sedative agent in TBI. It has the advantage of a relatively quick onset and offset of action facilitating neurological assessment. Clinicians should be mindful of the risk of PRIS, particularly when using >4 mg/kg/hour for >48 hours [23]. As an induction agent it may cause a fall in MAP and thus CPP, and this should be mitigated through the judicious use of vasopressors and fluid boluses. Propofol may be indicated in the treatment of refractory status epilepticus. Its use as an agent to achieve burst suppression may come at the expense of worsening haemodynamics.

## 3. Benzodiazepines

See Table 3. Benzodiazepines are commonly used as sedative agents in patients with TBI. They are nonselective CNS depressants that augment the action of GABA at GABA_A_ receptors, causing increased conductance of chloride ions. They have anxiolytic, amnesic, and anticonvulsant properties. Prior to the advent of propofol, midazolam was the most frequently used sedative in TBI in the UK [24], with lorazepam frequently being used in the US [25]. Midazolam offers the most benefits of the benzodiazepines for sedation in TBI, due to its shorter context sensitive *t*_1/2_ (2–2.5 hours) and faster onset and offset of action, compared to lorazepam (*t*_1/2_ 10–20 h) or diazepam (*t*_1/2_ 20–40 hours) [26]. It has a rapid onset as a result of high lipid solubility at physiological pH due to the closure of the imidazole ring. Its rapid hepatic metabolism accounts for its rapid offset of action [27] however some metabolites are active and accumulate with prolonged infusions. This may result in continued sedation even after drug cessation, particularly in the elderly or with liver impairment.

Whilst benzodiazepines reduce CBF, CMRO_2_, and ICP and increase seizure threshold, there is evidence that bolus doses significantly reduce MAP and CPP in severe TBI [28]. The depth of CMRO_2_ reduction possible with benzodiazepines is not as profound as barbiturates or etomidate, and burst suppression cannot be achieved [29].

Other disadvantages include significant respiratory depression and inhibition of the cough reflex, limiting its use in non-intubated patients. After prolonged sedation with benzodiazepines, tolerance develops, and on cessation, withdrawal symptoms including tremors, seizures, hypertension, and insomnia may occur, requiring ongoing longer acting benzodiazepines to be prescribed [30]. Benzodiazepines are a risk factor for ICU delirium [31], which is independently associated with poor outcomes [32].

There have been several studies comparing the safety and efficacy of benzodiazepines with other commonly used agents. In one RCT, 63 trauma patients, the majority with severe TBI, were randomised to receive either midazolam or 2% propofol infusions. Patients in both groups received morphine for analgesia. No significant difference in ICP or in wake-up time was demonstrated between the two groups. Similarly no significant differences were seen in haemodynamic variables between the two groups. Interestingly, there was a higher incidence of therapeutic failure in the propofol group either because of inadequate sedation or hypertriglyceridemia [33]. Other smaller, underpowered studies have also failed to demonstrate a difference in outcomes between these two agents [34].

Therefore benzodiazepines have a role in the sedation of patients where imminent neurological assessment is not required. They have significant disadvantages including an accumulation of metabolites, increasing tolerance with prolonged infusions, and an increased likelihood of delirium.

## 4. Narcotics

See Table 4. Opioid narcotics primarily have analgesic properties, and their sedative action may even be considered a side effect. However, various opioids are used in the sedation of patients with TBI, usually in combination with hypnotic agents to ensure analgesia and reduce hypnotic dose requirements. Analgesia-based protocols are feasible, with certain advantages over hypnotic (propofol and midazolam) sedative regimens [35]. Intravenous opioids used include morphine, fentanyl, sufentanil, and more recently remifentanil.

Opiates act on *μ*_1_ receptors (supraspinal analgesia), *μ*_2_ receptors (ventilatory depression, bradycardia, physical addiction), *κ* receptors (sedation, spinal analgesia), *ε* receptors (dysphoria, hallucinations, respiratory stimulation), and Δ receptors (analgesia, behavioural effects, and epileptogenic). The different opioids have variable effects on each receptor [26]. Opioids can produce hypotension by a number of mechanisms including a reduction in sympathetic tone and the stimulation of histamine release. This hypotension may be detrimental in patients with TBI in whom maintenance of cerebral perfusion pressure is vital.

Prior to the advent of newer agents morphine has been most commonly used as a narcotic in TBI. However, prolonged use of opioids such as morphine can lead to redistribution and accumulation, with potentially unpredictable delays in awakening. The *t*_1/2_ of morphine is increased in renal failure, as a proportion of both the parent drug and an active metabolite, morphine-6-glucuronide, are excreted renally [36]. In addition, tachyphylaxis can lead to increasing dose requirements with subsequent withdrawal phenomena and a possible rebound increase in ICP on cessation.

Shorter acting opioids include fentanyl, alfentanil, sufentanil, and remifentanil. These are more lipid soluble than morphine and so have a faster onset of action [37]. Metabolism to inactive metabolites leads to less accumulation in renal failure. Nevertheless, with prolonged infusion shorter acting opioids can accumulate and impede neurological assessment. For example, with an increasing duration of fentanyl infusion, saturation of inactive tissue sites and a return of opioid from peripheral compartments mean that there is a prolonged context-sensitive half time relative to sufentanil.

Studies of the effects of opioids on ICP have been inconsistent. However, there is evidence that the administration of high bolus doses of opioids may have potentially deleterious effects in TBI, with some studies showing an increase in ICP and a fall in CPP. These effects occurred despite controlling PaCO_2_. Interestingly, in those studies that prevented hypotension, an increase in ICP was not seen. It is suggested that hypotension may increase ICP and decrease CPP through cerebral autoregulatory reflexes [9]. It is unclear to what extent opioids may induce seizure activity. Whilst there are numerous case reports of clinical seizure activity, it has been argued that many of these represent muscle rigidity associated with high doses of opioid rather than seizure activity per se [38].

There has been increased interest in remifentanil as an alternative opioid sedative in TBI. Remifentanil is a potent, synthetic opioid receptor agonist, which differs from other synthetic opioids in that it undergoes rapid hydrolysis by tissue and plasma esterases. This rapid metabolism and lack of accumulation facilitate faster waking and neurological assessment of patients with TBI [39]. An RCT on neuro-intensive care patients showed analgesia-based sedation with remifentanil offered faster and more predictable time to assessment of neurological function than a hypnotic-based technique (propofol or midazolam) [40]. Furthermore, remifentanil was well tolerated in patients with TBI, with a significantly shorter time to extubation in patients who had received remifentanil compared with patients who had received morphine [11].

Opioids have a role as an adjunct to other sedative agents, for example in combination with propofol. They may reduce sedative requirements of other agents and provide effective analgesia and anxiolysis. Prolonged infusions of opioids, particularly morphine, may accumulate and hinder neurological assessment. When opioids are administered as a bolus, there is a risk of increasing the ICP, particularly when the MAP is allowed to fall.

## 5. Barbiturates

See Table 5. Barbiturates, particularly pentobarbital and thiopentone, have previously played a central role in the sedation of patients with TBI [41]. However, with the advent of newer agents with less disadvantages, thiopentone is largely confined to use as an induction agent, for the treatment of refractory elevated ICP and for status epilepticus. Barbiturates stimulate *γ*-aminobutyric acid (GABA) receptors and inhibit *α*-amino-3-hydroxy-5-methyl-4-isoxazolepropionic acid (AMPA) receptors in the CNS producing dose-dependent sedation and general anaesthesia.

High lipid solubility allows rapid transfer across the blood-brain barrier and exceptionally fast onset of action. The induction of anaesthesia sufficient for intubation within one arm-brain circulation time initially popularized the use of thiopentone as an induction agent in rapid-sequence intubation (RSI) [42]. The hypotensive effects caused by direct myocardial and central vasomotor depression should be anticipated and addressed by using only low doses and coadministering vasopressors such as metaraminol or phenylephrine if the blood pressure is suboptimal before RSI.

A recent Cochrane review concluded that barbiturates are not indicted as a maintenance sedative agent or for use prophylactically to prevent elevations in ICP [43], predominantly because the hypotension and other side effects offset any ICP lowering effect on CPP.

Significant accumulation will occur with repeated doses or infusions due to the long context-sensitive *t*_1/2_ and the elimination kinetics changing from 1st to zero order at plasma levels >30 mg/L. To treat refractory elevated ICP or refractory status epilepticus, a clinical endpoint of burst suppression on EEG is targeted, which requires plasma levels >40 mg/L. Unfortunately, the high doses of thiopentone required to achieve this preclude neurological assessment for several days.

Therefore thiopentone may be used as an induction agent in TBI if hypotension is not already problematic and precautions are taken. It has a role in treatment of refractory elevated ICP and refractory status epilepticus, but not as a maintenance sedative in TBI.

## 6. Etomidate

See Table 6. Etomidate is a carboxylated imidazole derivative predominantly used as an intravenous induction agent in the setting of haemodynamic instability. It causes less hypotension and cardiovascular depression than other sedatives in this context [44], with the exception of ketamine. Other advantages include a rapid onset of anaesthesia (10s) lasting 3–5 minutes following a dose of 0.3 mg/kg, and a short elimination *t*_1/2_ of 2.6 h [45]. There is a reduction in CBF and ICP [46] and it can even achieve burst suppression on EEG [47].

However, the safety of etomidate has been questioned. Continuous infusions have been shown in a retrospective study to cause a significant increase in mortality [48]. Etomidate causes adrenal suppression by suppressing corticosteroid synthesis through the inhibition of the enzyme 11-*β*-hydroxylase, which converts 11-deoxycortisol to cortisol. This effect has been demonstrated with both infusions and with a single bolus. A single dose of etomidate reduces the synthesis of cortisol and aldosterone and increases the risk of relative adrenocortical insufficiency (RAI) for at least 24 hours [49]. Hypotension related to RAI has implications for CPP and neurological outcome. Etomidate may also lower seizure threshold [50]. Other adverse effects include pain on injection, myoclonic movements, and nausea and vomiting [51].

Therefore etomidate should be avoided as a continuous sedative agent in TBI but may be considered with caution as an induction agent, although ketamine offers many of the same advantages without the risks of adrenal suppression.

## 7. Ketamine

See Table 7. Ketamine is an N-methyl-D-aspartate receptor antagonist. It has traditionally been avoided in the management of patients with traumatic brain injury owing to concerns that it may increase intracranial pressure. Furthermore, there are theoretical concerns regarding its epileptogenic potential. Indeed, it receives little attention in guidelines for the management of TBI [1]. Conversely, it has been argued that in comparison to most widely used sedative agents ketamine does not decrease blood pressure and therefore may preserve cerebral perfusion pressure. In particular, it has been argued that this haemodynamic stability enables ketamine to be used as a safe induction agent in patients with TBI [52].

Concerns regarding the potential for ketamine to raise ICP stem from small case control series several decades ago in patients with abnormal CSF flow dynamics [53]. A rise in ICP was observed in spontaneously breathing patients, undergoing diagnostic pneumoventriculography, in whom ketamine was administered to. However, this rise in ICP only occurred in those patients with abnormal CSF pathways. In the remaining patients there was an overall rise in MAP, an increase in cerebral blood flow, and improved cerebral perfusion pressure [54, 55].

Several recent studies have refuted the original findings and showed no statistically significant rise in ICP in brain injured patients who are sedated with ketamine [56]. Bourgoin et al. randomised patients with TBI to receive either sufentanyl-midazolam or ketamine-midazolam sedation using target controlled infusions. The target concentrations of sufentanil and ketamine were doubled for 15 minutes, and the plasma concentrations of both were measured. There was no significant change in ICP or CPP with increased plasma concentrations. In an interesting editorial, the possibility that cerebral haemodynamics are better preserved through the use of target controlled infusion was discussed [57]. Whilst bolus doses of some commonly used sedatives may adversely affect haemodynamics and increase ICP, it is argued that a system relying on pharmacokinetic models alone is insufficient in managing patients with TBI.

Another study looked at the use of ketamine in 30 sedated and ventilated children with TBI and raised ICP resistant to first-tier therapies [58]. Variables examined included ICP, hemodynamic variables, and CPP. Ketamine was administered as a single dose of 1–1.5 mg/kg either to prevent further ICP increases during distressing procedures or as an additional measure to lower ICP. There was an overall decrease in ICP and increase in CPP in both situations. The authors conclude that ketamine is a safe and effective sedative agent to use in patients with TBI.

There is conflicting data as to whether ketamine induces epileptiform activity. The blocking of NMBA receptors and subsequent entry of calcium into neurons may limit seizure activity. Furthermore, the use of ketamine as an adjunct in the treatment of status epliepticus is well described in the literature [59]. The antagonism of NMDA receptors decreases the release of neurotoxic glutamate and may impart a protective effect in patients with traumatic brain injury [60].

Therefore ketamine is indicated particularly as an induction agent in patients with TBI and haemodynamic instability. It may have a role for refractory seizure activity.

## 8. Dexmedetomidine

See Table 8. Dexmedetomidine is a highly selective alpha-2 receptor agonist that acts by a receptor distinct from the GABA receptor utilised by propofol and the benzodiazepines. A high selectivity for alpha-2 receptors, seven to eight times that of clonidine, explains its anxiolytic and sedative effects. A relatively short elimination *t*_1/2_ of two hours enables intravenous titration to effect. Furthermore, dexmedetomidine does not appear to cause respiratory depression, with one study reporting no significant difference in respiratory rate and oxygen saturations between dexmedetomidine recipients and those that received placebo. This enables it to be continued after-extubation [61, 62]. Hypotension and bradycardia are among the most commonly reported side effects of dexmedetomidine, particularly when using a loading dose. For this reason, some commentators recommend an avoidance of a loading dose in patients with TBI.

Several trials have examined the use of dexmedetomidine sedation in ICU patients.

Riker et al. performed a prospective, double-blinded RCT in medical and surgical ICU patients comparing the efficacy and safety of dexmedetomidine with midazolam sedation [63]. Patients in the dexmedetomidine arm spent less time on the ventilator and experienced less hypertension and tachycardia. 42.2% of patients in the dexmedetomidine arm experienced bradycardia compared to 18.9% of patients who received midazolam sedation.

A potential advantage of dexmedetomidine may be in decreasing the incidence or severity of delirium. Many commonly used sedatives, including opioids and benzodiazepines, have been shown to increase the risk of delirium. In one prospective, double-blinded RCT, patients after cardiac surgery were randomised to receive either a dexmedetomidine or morphine-based sedative regimen [64]. Patients in the dexmedetomidine arm showed a significant reduction in the duration of delirium, although there was no statistically significant reduction in the incidence of delirium. A reduction in the incidence of delirium was also found in an a priori subgroup analysis of the MENDS trial. There was a reduced duration of brain dysfunction, particularly in septic patients [58].

There have been relatively few studies examining the role of dexmedetomidine in patients with TBI. Its use in neurosurgical patients was described in a retrospective study by Aryan et al. [65]. They describe a mean increase in cerebral perfusion pressure and a decrease in intracranial pressure in the 39 patients studied. The relatively small sample size and retrospective nature of this study limit its conclusions, and the authors argue for further studies to establish an optimal dosage regimen in neurosurgical patients. Grof et al. undertook a small, prospective, observational study, of patients receiving dexmedetomidine on a neurosurgical ICU [66]. The majority of these patients had traumatic brain injury. Dexmedetomidine was utilised in an attempt to wean patients off other sedative regimens. Relatively high doses of dexmedetomidine were required to achieve the desired level of sedation, up to a rate of 2.5 mcg/kg/hour. The authors postulate that significant changes in neurotransmitter systems in TBI might explain the need for higher doses of dexmedetomidine in this patient population.

There is a need for further high-quality RCTs to evaluate the use of dexmedetomidine as a sedative agent both in general ICU patients and in patients with TBI. The SPICE pilot study will examine the feasibility of conducting a large multi-centre trial, comparing current sedation practice with a dexmedetomidine-based sedation regimen. The DahLIA trial is currently recruiting patients and is a prospective, double-blinded RCT comparing dexmedetomidine to placebo in the treatment of delirium and agitation.

Therefore dexmedetomidine has a number of potential advantages as a sedative agent in TBI. There is evidence that it may reduce delirium and a lack of respiratory depressant effects enables it to be used in non-intubated patients.

## 9. Conclusion

Sedation is a vital component of the management of patients with traumatic brain injuries. However, there is limited high-quality evidence examining outcomes in TBI to guide clinicians on their choice of agent. Consequently a wide variety of agents and dosages are used. Recent work has challenged traditional views on the best agents to use in TBI. For example, there is increasing evidence that ketamine may be safe to use in TBI as an induction agent and has advantages over traditional agents such as the barbiturates. There has also been increased interest in shorter acting, newer agents such as remifentanil and dexmedetomidine. These offer potential advantages in allowing faster recovery of consciousness and assessment of neurology in patients.

There is a need for further prospective, randomised controlled trials, examining both physiological and clinical outcomes, to assess these agents in the context of traumatic brain injury. Meanwhile, in the absence of extensive high-quality evidence to guide clinicians in their choice of agent, there is a need for a pragmatic approach, based on the clinical situation and knowledge of the potential advantages and disadvantages of each agent.

## 10. Recommendations

See Table 9. For induction of anaesthesia in TBI, there is no single agent that is perfect and the way in which the medication is given, and the dose used, may be more important than the agents themselves. Considered preparation, experienced assistants and meticulous control and monitoring of blood pressure are essential. Thiopentone remains a reasonable choice, with the proviso that it is used judiciously in the haemodynamically unstable patient. Alternative induction agents include propofol (usually requiring a concomitant vasopressor bolus) or ketamine. There is little role for etomidate either as an agent for induction or continued sedation.

Propofol as an agent for continued sedation, usually administered with a short-acting narcotic, offers the advantage of a relatively rapid offset of sedation, facilitating neurological assessment. Remifentanil has many advantages over other narcotics in this setting as long as hyperalgesia on cessation is considered. In patients who require high doses of propofol, hypotensive patients, or for more prolonged sedation, midazolam is a suitable alternative. Thiopentone is not indicated as a maintenance sedative agent in TBI, and its use is primarily limited to the treatment of refractory intracranial hypertension. Dexmedetomidine shows promise as a sedative agent in TBI, particularly in the non-intubated patient.

Thanks to Professor Richard Lee for his helpful suggestions regarding this review.

## Figures and Tables

**Table 1:** 

Abbreviations and explanations	
(i) AMPA: *α*-amino-3-hydroxy-5-methyl-4-isoxazolepropionic acid, GABA: *γ*-Aminobutyric acid (ii) EEG: Electroencephalogram (iii) CMRO_2_: Cerebral Metabolic Rate of Oxygen (iv) CPP: Cerebral Perfusion Pressure (v) ICP: Intracranial Pressure (vi) IV: Intravenous (vii) MAP: Mean Arterial Pressure (viii) *t*_1/2_: Half-life (ix) *Context sensitive t*_1/2_: the time taken for blood plasma concentration of a drug to decline by one half after an infusion designed to maintain a steady state (i.e., a constant plasma concentration) has been stopped. The “context” is the duration of infusion [5].	

**Table 2:** 

Propofol	
Group	Phenol Derivative
Mechanism of Action/Pharmakodynamics	Potentiation GABA_A_ receptors Na^+ ^channel blocker

Neuroprotective effects	Reduces CBF, CMRO_2_ and ICP Reduces MAP, therefore variable effect on CPP Increases seizure threshold

Pharmacokinetics	Rapid hepatic metabolised, with extra-hepatic metabolism *t*_1/2_ 2–24 hours, but rapid peripheral distribution Short context sensitive *t*_1/2_

Advantages	Favourable effects on CBF, CMRO_2 _and ICP Rapid onset of action Relatively short context sensitive *t*_1/2_ facilitating neurological assessment

Disadvantages and major side effects	Hypotension may worsen CPP High lipid load Associated with elevated liver enzymes & pancreatitis Potential for PRIS, particularly with prolonged, high dose infusions Formulation may support bacterial and fungal growth Contraindicated if allergic to egg or soybeans

Dosage	Induction: 1–2.5 mg/kg, 0.5–1.5 mg/kg in elderly or limited cardiovascular reserve Maintenance of sedation: 1.5–4.5 mg/kg/hour, titrated to desired effect

Other significant facts	Increased risk of PRIS at infusions >4 mg/kg/h for >48 h

Appropriate roles in TBI	Induction agent, caution in hypotension Continuous infusion to provide sedation in TBI Refractory elevated ICP Refractory seizures

**Table 3:** 

Midazolam	
Group	Imadobenzodiazepine
Mechanism of Action/Pharmakodynamics	GABA_A_ receptor agonist Chloride channel activation, Kappa opioid agonist

Neuroprotective effects	Reduces CBF, CMRO_2_ and ICP but minimal effect beyond that of sedation Reduces MAP, variable effect on CPP Raises seizure threshold

Pharmacokinetics	Onset of action 2–4 minutes 94% protein bound Highly lipid soluble Hepatic metabolism Renal excretion (some bile) Short context sensitive *t*_1/2_ (2.4 h)

Advantages	Shorter *t*_1/2_ than other benzodiazepines Causes less hypotension than barbiturates or propofol

Disadvantages and major side effects	Metabolites accumulate delaying neurological assessment post cessation of infusion Boluses in TBI reduce MAP (and CPP) Withdrawal syndrome Delirium Respiratory and cough reflex suppression Tachyphylaxis after 72 hours Plateau effect on reducing ICP, where increasing doses have no effect

Dosage	Induction: 0.1 mg/kg Maintenance of sedation: 0.01–0.2 mg/kg/hour

Other significant facts	Interaction with peripheral benzodiazepine leucocyte receptors so may have immunosuppressant effect

Appropriate roles in TBI	Induction of anaesthesia Maintenance of sedation in hypotensive patients with TBI Maintenance of sedation when imminent neurological assessment not required Treatment of seizures

**Table 4:** 

	Morphine	Fentanyl	Alfentanil	Sufentanil	Remifentanil
Pharmacodynamics	*μ*_1_, *μ*_2_, *κ* and Δ agonists				

Elimination *t*_1/2_ (h)	3	3.7	1.5	2.2	0.25

Distribution *t*_1/2_	3–11 min	10–30 min	15 min	5 min	1 min

Neuroprotective effects	May increase ICP	Minimal effect beyond the analgesic effect on CBF and CMRO_2_			

Pharmacokinetics	Onset 6 min Peak effect 20 min (IV) 30% protein bound Hepatically metabolised to active metabolites Renal clearance	95% protein bound High lipid solubility 75% first pass pulmonary uptake Hepatically metabolised to active metabolites Renal clearance	Onset Peak 90 s Duration 5–10 min 90% protein bound Hepatically metabolised Renal clearance	Hepatically metabolised Renal clearance	Peak 60 s Small Vd Rapid clearance Rapid ester hydrolysis by plasma esterases to inactive metabolite (Independent of renal & hepatic function)

Advantages	Lower cost Relative haemodynamic stability Hypnotic agent sparing Analgesic properties	Lower cost Relative haemodynamic stability Hypnotic agent sparing Analgesic properties	Relative haemodynamic stability Hypnotic agent sparing Analgesic properties	Relative haemodynamic stability Hypnotic agent sparing Analgesic properties	Very rapid onset/offset Less nausea Relative haemodynamic stability Hypnotic agent sparing Analgesic properties

Disadvantages and major side effects			Hypotension Bradycardia Respiratory depression Cough reflex suppression Seizures Rigidity Constipation Spasm sphincter of Oddi Nausea Pruritis		

Dosage	0.05–0.1 mg/kg/hr	Induction: 1–3 mcg/kg Maintenance: 0.5–2 mcg/kg/h	Induction: 10–50 mcg/kg Infusion: 0.5–1 mcg/kg/min	Induction: 4 mcg/kg	Bolus: 1 mcg/kg Infusion: 0.0125–1 mcg/kg/min

Appropriate uses in TBI	Long term analgesia Palliation	Co-Induction agent Continuous infusion Palliation	Co-Induction agent	Co-Induction agent	Co-Induction agent Continuous infusion infusion

**Table 5:** 

Thiopentone	
Group	Barbiturate
Mechanism of Action/Pharmacodynamics	Stimulate GABA receptors Inhibit AMPA receptors

Neuroprotective effects	Reduces CBF, CMRO_2 _and ICP Reduces MAP, therefore variable effect on CPP Raises seizure threshold

Pharmacokinetics [6]	Hepatically metabolised 0.5% renal excretion unchanged Elimination *t*_1/2_ 11.6 h First to zero order kinetics if plasma high Significant accumulation

Advantages	Rapid onset of action as induction agent Favourable effects on CBF, CMRO_2 _and ICP Inexpensive

Disadvantages and major side effects	Accumulation with prolonged infusion Hypotension Gastroparesis Loss of thermoregulation Immunosuppression Hypokalaemia during infusion Hyperkalaemia on emergence Life threatening arrhythmias on coma emergence

Dosage	Induction of anaesthesia: 2–5 mg/kg EEG burst suppression: 40 mg/kg followed by infusion at 4–8 mg/kg/h, titrated to EEG

Other significant facts	May precipitate if given concurrently with IV muscle relaxants [7]

Appropriate uses in TBI	Induction of anaesthesia, with caution regarding hypotension Refractory elevated ICP Refractory status epilepticus

**Table 6:** 

Etomidate	
Group	Caroboxylated imidazole derivative
Mechanism of Action/Pharmakodynamics	GABA_A_ receptor agonist

Neuroprotective effects	Reduces CBF, CMRO_2_ and ICP Maintains or increases CPP Lowers seizure threshold

Pharmacokinetics	75% protein bound Highly lipid soluble High volume of distribution, three compartment model Hepatic metabolism Renal excretion (some bile) Short context sensitive *t*_1/2_ (4.8 h)

Advantages	Rapid onset of action as induction agent Only lasts 3–5 minutes after single bolus Favourable effects on CBF, CMRO_2_ and ICP

Disadvantages and major side effects	Adrenal suppression Metabolic acidosis from propylene glycol vehicle Pain on injection Myoclonic movements Nausea and vomiting

Dosage	Induction: 0.2–0.4 mg/kg

Other significant facts	Originally developed as an anti-fungal agent

Appropriate uses in TBI	Induction of anaesthesia, with caution regarding adrenal suppression

**Table 7:** 

Ketamine	
Group	Phencyclidine derivative
Mechanism of Action/Pharmacodynamics	Competitive NMDA receptor antagonist Interaction with opioid and muscarinic receptors Na^+^ Channel

Effect on ICP	None or decrease

Neuroprotective effects	Decreased glutamate

Pharmacokinetics	20% Bioavailability 40% protein bound Distribution *t*_1/2 _10 minutes Hepatic metabolism Elimination *t*_1/2_ 2.5 h

Advantages	Preserves MAP and CPP

Disadvantages and major side effects	Early studies ↑ICP, ?epileptogenic Hallucinations/Emergence phenomena

Dosage	Induction: 2 mg/kg Maintenance: 50 mcg/kg/min

Other significant facts	

Appropriate uses in TBI	Haemodynamic instability

**Table 8:** 

Dexmedetomidine	
Group	Selective *α*2 adrenergic agonist
Mechanism of Action/Pharmacodynamics	Peripheral *α*2A, brain & spinal cord *α*2B, *α*2C adrenoreceptor subtypes

Neuroprotective effects	Reduces CBF and ICP

Pharmacokinetics	Hepatic metabolism Distribution *t*_1/2_ 6 minutes Elimination *t*_1/2_ 2 hours

Advantages	Minimal respiratory depression Reduction in delerium

Disadvantages and major side effects	Hypotension (28%) Bradycardia Arrhythmias including atrial fibrillation Relatively high cost

Dosage	Loading dose: 1 mcg/kg Infusion: 0.42–1.0 mcg/kg/hour

Other significant facts	Minimal effect on respiratory function

Appropriate uses in TBI	Maintenance sedation agent pre & post extubation Management of agitated delirium

**Table 9:** 

Induction agents	
(i) Haemodynamically unstable	Ketamine (2 mg/kg) OR Midazolam (0.1 mg/kg) and fentanyl (1–3 mcg/kg)
(ii) Haemodynamically stable	Thiopentone (1–3 mg/kg OR propofol (0.5–2.5 mg/kg), with fentanyl (1–3 mcg/kg)

Maintenance agents	Propofol (1.5–4.5 mg/kg/h) and fentanyl (0.5–2 mcg/kg/h)
